# The Antimicrobial Peptide CRAMP-34 Eradicates *Escherichia coli* Biofilms by Interfering with the *kduD*-Dependent Network

**DOI:** 10.3390/antibiotics15010083

**Published:** 2026-01-14

**Authors:** Hongzao Yang, Jing Xiong, Sisi Su, Zhuo Yang, Wu Yang, Lianci Peng, Suhui Zhang, Jinjie Qiu, Yuzhang He, Hongwei Chen

**Affiliations:** 1 College of Veterinary Medicine, Southwest University, Chongqing 402460, China; yhz03008@swu.edu.cn (H.Y.); 1347845194@qq.com (J.X.); 17737435386@163.com (S.S.); 2327540095@qq.com (Z.Y.); penglianci@swu.edu.cn (L.P.); 365596255@qq.com (Y.H.); 2 National Center of Technology Innovation for Pigs, Chongqing 402460, China; 446116409@qq.com; 3 Chi Institute of Traditional Chinese Veterinary Medicine, Southwest University, Chongqing 402460, China; 429482286@qq.com; 4 Dali Jinming Animal Pharmaceutical Co., Ltd., Dali 671005, China; 380750440@qq.com; 5 Chongqing Academy of Animal Sciences, Chongqing 402460, China

**Keywords:** *Escherichia coli*, *kduD*, biofilm, CRAMP-34, eradicate

## Abstract

**Background/Objectives**: Bacterial biofilms formed by *Escherichia coli* pose a significant challenge in veterinary medicine due to their intrinsic resistance to antibiotics. Antimicrobial peptides (AMPs) represent a promising alternative. AMPs exert their bactericidal activity by binding to negatively charged phospholipids in bacterial membranes via electrostatic interactions, leading to membrane disruption and rapid cell lysis. **Methods**: In vitro assays including MIC determination, biofilm eradication testing (crystal violet, colony counts, and CLSM), swimming motility, and EPS quantification were performed. CRISPR/Cas9 was used to construct and complement a *kduD* mutant. A transposon mutagenesis library was screened for biofilm-defective mutants. In an in vivo murine excisional wound infection model treated with the mouse cathelicidin-related antimicrobial peptide (CRAMP-34), wound closure and bacterial burden were monitored. Gene expression changes were analyzed via RT-qPCR. **Results:** CRAMP-34 effectively eradicated pre-formed biofilms of a clinically relevant, porcine-origin *E. coli* strain and promoted wound healing in the murine infection model. We conducted a genome-wide transposon mutagenesis screen, which identified *kduD* as a critical gene for robust biofilm formation. Functional characterization revealed that *kduD* deletion drastically impairs flagellar motility and alters exopolysaccharide production, leading to defective biofilm architecture without affecting growth. Notably, the anti-biofilm activity of CRAMP-34 phenocopied aspects of the *kduD* deletion, including motility inhibition and transcriptional repression of a common set of biofilm-related genes. **Conclusions:** This research highlights CRAMP-34 as a potent anti-biofilm agent and unveils *kduD* as a previously unrecognized regulator of *E. coli* biofilm development, which is also targeted by CRAMP-34.

## 1. Introduction

*Escherichia coli* (*E. coli*) causes persistent diarrhea and antimicrobial resistance due to its propensity to form aggressive and dense bacterial biofilms [1]. The bacteria in biofilms secrete various components, such as extracellular polymeric substances (EPSs) that cause bacterial accumulation, forming multiple layers, which protect the embedded cells from antimicrobial agents and host immune defenses; these infections are called biofilm-associated infections (BAIs) [2,3]. Previous studies have reported that the tolerance of biofilms to various antibiotics is 10–1000 times greater than that of planktonic cells [4]. The life cycle of biofilms includes the following stages: reversible/irreversible adhesion, formation, maturation, and dispersion [5]. In the initial attachment stage, flagella and fimbriae facilitate mechanical attachment to the surface. As the cell density increases, the autoinducer concentration reaches a threshold, and the autoinducer/regulatory protein complex induces or represses the expression of target genes [6]. Pili-mediated twitching motility and a high concentration of cyclic diguanylic acid (c-di-GMP) enable the attached cells to aggregate to form microcolonies that then produce EPSs, thereby ensuring the adherence of the bacteria to the surface of a highly complex bio-molecular layer [7]. Fully mature biofilms typically have a three-dimensional mushroom-like structure [8]. These highly structured biofilms are difficult to eradicate.

Genes that are directly involved in *E. coli* biofilm formation include the synthetic operon pgaABCD for Poly-β-1,6-N-acetyl-d-glucosamine (poly-β-1,6-GlcNAc; PGA), glycogen synthesis operon *glgCAP*, autoaggregation adhesin Artigen 43, *wza* gene for synthesizing the multimeric outer membrane protein complex, operons *bcsABZC* and *bcsEFG* that encode the cellulose synthase complex, and operons *csgBAC* and *csgDEFG* that are involved in curli fimbria protein synthesis and assembly [9,10,11]. Another study showed that more than 60 genes are directly involved in the regulation of *E. coli* biofilm formation via signal transduction pathways, such as the quorum sensing (QS) system, second messenger c-di-GMP system, two-component regulatory system (TCS), transcription regulatory protein pathways, and non-coding small RNA (sRNA) pathways [12,13,14]. Nonetheless, it is not known if there are other genes that regulate E. coli biofilm formation.

Our studies have shown that the mouse cathelicidin-related antimicrobial peptide (CRAMP-34) inhibits biofilm formation and can eradicate mature biofilms formed by the biofilm model strain *Pseudomonas aeruginosa* [15]. However, whether CRAMP-34 has the same inhibitory effect on *E. coli* biofilms has yet to be clarified. In addition, the key regulatory genes for biofilm formation and maturation in *E. coli* also need to be explored further. Therefore, the current study focused on a strain of *E. coli* isolated from a porcine source that has a strong biofilm formation ability and carries the mobilized colistin resistance (*mcr*) gene. Despite the well-characterized roles of operons such as *bcsABZC* and *csgDEFG* in biofilm formation, the complete genetic network regulating this process in *E. coli* remains incompletely understood [16,17]. To systematically identify novel regulators, we employed a functional screen using transposon mutagenesis. This approach, combined with subsequent CRISPR/Cas9-mediated validation, led to the identification of an previously unknown key gene, *kduD*, which encodes 2-dehydro-3-deoxy-D- gluconate 5-dehydrogenase (*KduD*), as a critical regulator of biofilm formation in this strain. Furthermore, given the central role of *KduD*, we investigated its potential as a target for anti-biofilm agents and specifically evaluated whether it mediates the anti-biofilm effect of the antimicrobial peptide CRAMP-34.

## 2. Results

### 2.1. CRAMP-34 Demonstrates Potent Biofilm-Eradicating Activity and Promotes Wound Healing In Vivo

**Figure 1:**
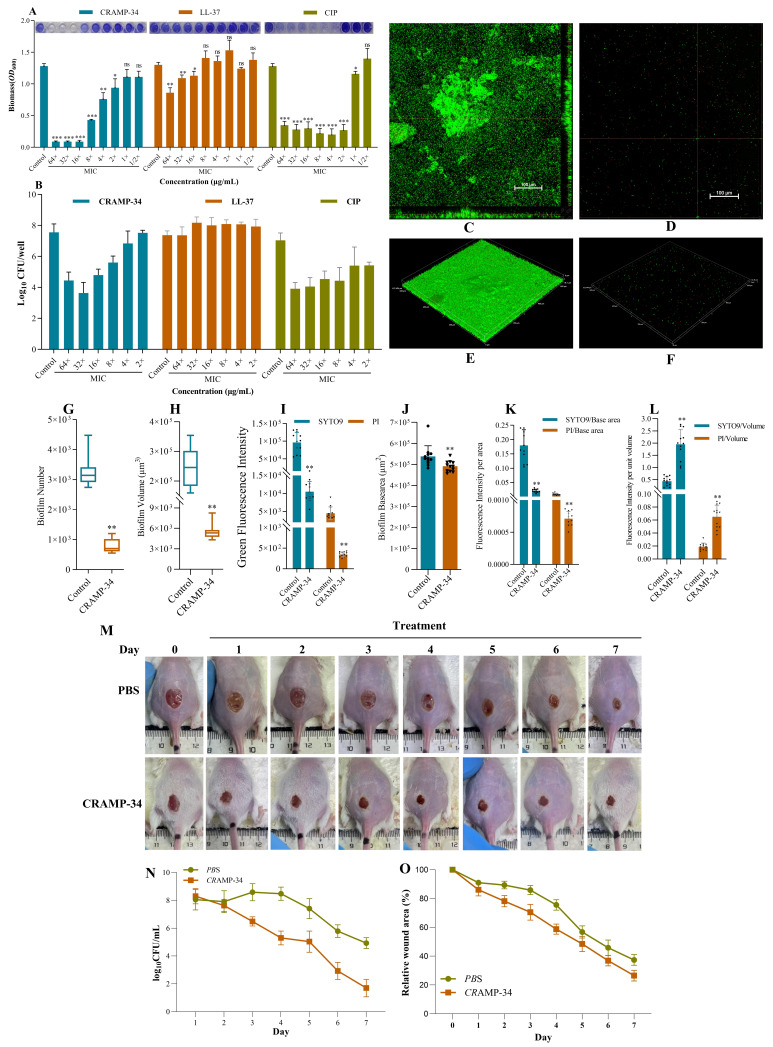
CRAMP-34 demonstrates potent biofilm-eradicating activity and promotes wound healing in vivo. (**A**) The biomass of Ec032 biofilms (1-day-old) treated with CRAMP-34, LL-37, and CIP was measured using crystal violet staining. (**B**) The viable bacteria of Ec032 biofilms (1-day-old) treated with CRAMP-34, LL-37, and CIP were detected by crystal violet staining. (**C**,**D**) The orthogonal views and three-dimensional representations of the biofilms in the control group under a 20× objective. (**E**,**F**) The orthogonal views and three-dimensional representations of the biofilms treated with CRAMP-34 using a 20× objective. (**G**) Biofilm cell number. (**H**) Biofilm volume. (**I**) The fluorescence intensity of live and dead bacteria. (**J**) Biofilm base area. (**K**) The fluorescence intensity per unit area (fluorescence intensity/base area). (**L**) Fluorescence intensity per unit volume (fluorescence intensity/volume). (**M**) Representative photos of wounds of mice from different treatment groups. (**O**) Antibacterial efficacy of different treatments based on the results of bacterial colony counts. (**N**) Statistics for wound shrinkage rates. Note: The MIC values of CRAMP-34, LL-37, and CIP were 7.8125 μg/mL, 15.625 μg/mL, and 0.015625 μg/mL, respectively. * *p*  <  0.05, ** *p*  <  0.01, and *** *p*  <  0.001.

Different concentrations of CRAMP-34 were used on 1-day-old preformed biofilms; ciprofloxacin (CIP) and human antibacterial peptide LL-37 were used as controls. The MIC values of CRAMP-34, LL-37, and CIP were 7.8125 μg/mL, 15.625 μg/mL, and 0.015625 μg/mL, respectively. The results of the crystal violet staining showed that CRAMP-34 and CIP significantly reduced the biomass of *Ec*032 biofilms at a concentration of 4–64× MIC. However, CRAMP-34 exerted a significant effect on reducing the biomass of Ec032 biofilms at 16× MIC, with a rate of 92.95% (Figure 1A). The results of the colony-counting assay showed that CRAMP-34 killed more biofilms than LL-37 and CIP; the biofilms decreased by 2.76 log values with a killing rate of 99.83% at 16× MIC (Figure 1B).

To visualize the anti-biofilm activity of CRAMP-34, SYTO 9 (living) and propidium iodide (PI) (dead) staining was performed and analyzed using CLSM. As shown in Figure 1 C–F, the CRAMP-34 intervention group biofilms were significantly reduced compared to *Ec*032. The results showed that CRAMP-34 remarkedly reduced *Ec*032 biofilms at a concentration of 125 µg/mL, decreasing the cell number (75.90%), volume (97.69%), and base area (8.69%) (Figure 1H–I). Furthermore, the fluorescence intensity of SYTO 9- and PI-positive bacteria decreased by 89.11% and 92.28%, respectively (Figure 1J). In addition, the ratio of fluorescence intensity of SYTO 9 and PI per unit base area of the biofilms decreased by 88.09% (*p* < 0.01) and 91.63% (*p* < 0.01), respectively (Figure 1K), and the ratio of fluorescence intensity of SYTO 9 and PI per unit volume of the biofilms increased by 77.68% (*p* < 0.01) and 70.91% (*p* < 0.01), respectively (Figure 1L).

The wounds of each group of mice were photographed regularly to observe the healing process. As shown in Figure 1M, compared with the PBS group, the wound healing rate in the CRAMP-34 treatment group was significantly accelerated, and the wound area was significantly reduced. Residual bacteria at the wound site were sampled daily and counted using the plate method. Compared with the PBS group, the number of bacterial colonies in the CRAMP-34 treatment group was significantly reduced (Figure 1N) and the relative wound closure rate became increasingly faster (both differences were statistically significant), which further verified the antibacterial effect of CRAMP-34 (Figure 1O). In summary, CRAMP-34 significantly promoted the healing of wounds caused by *E. coli*.

### 2.2. A Genome-Wide Screen Identifies kduD as a Novel Essential Gene for Robust Biofilm Formation

**Figure 2:**
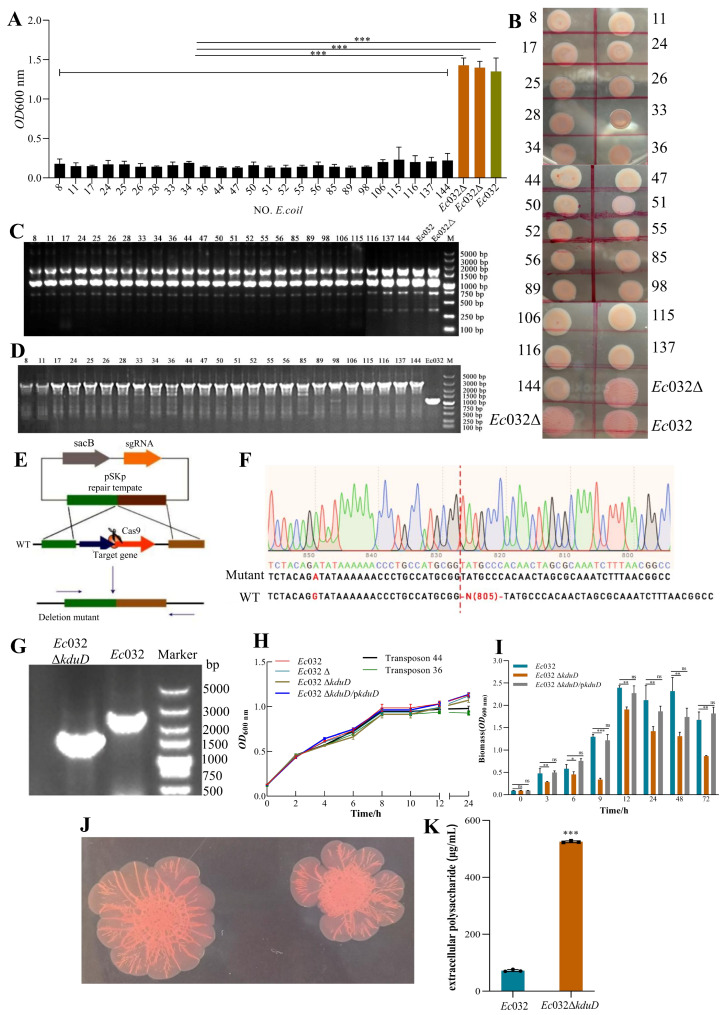
Identification of kduD through transposon mutagenesis and its role in biofilm formation, colony morphology, and EPS production in Ec032. (**A**) Biomass of mutant biofilms (1-day-old) in 96-well plates was measured using crystal violet staining. (**B**) Congo red staining showing colony morphology of Ec032-related mutant strains. (**C**) Electrophoretogram of ERIC-PCR products. (**D**) Identification of transposon insertion sites in mutant strains. (**E**) Schematic diagram of the pCasKp-OriT/pSGKp system construction process. (**F**) Peak map from Sanger sequencing. (**G**) Electrophoretogram. (**H**) Growth curves of various bacterial strains. (**I**) Biomass (1-day-old) was stained in a test tube with crystal violet. (**J**) Congo red staining showing colony morphology. (**K**) The EPS content at the same bacterial density. * *p*  <  0.05, ** *p*  <  0.01, and *** *p*  <  0.001.

An *Ec*032 random Mariner transposon mutagenesis library comprising 141,667 mutants was constructed to identify genes involved in biofilm formation. From this library, 25 independent mutants exhibiting a pronounced reduction in biofilm formation were isolated (Figure 2A). Subsequent phenotypic characterization via the Congo red assay revealed that these mutants collectively displayed a loss of colony wrinkling, a transition to a smoother morphology, and diminished pigmentation compared to the wild-type *Ec*032 and an empty vector control strain (*Ec*032Δ) (Figure 2B). ERIC-PCR analysis confirmed that all 25 strains were genuine transposon mutants, ruling out contamination (Figure 2C). Whole-genome sequencing and comparative analysis pinpointed the transposon insertion site in all the mutants to a single specific locus, which we designated as *kduD* (Figure 2D).

To definitively establish the role of *kduD* in biofilm formation, an in-frame deletion mutant (*Ec*032Δ*kduD*) was generated using the CRISPR/Cas9 system (Figure 2E), with the deletion verified by Sanger sequencing and electrophoretic analysis (Figure 2F,G). The deletion of *kduD* had no discernible impact on bacterial growth kinetics (Figure 2H). However, quantitative assessment of biofilm formation using crystal violet staining demonstrated a significant impairment in the *Ec*032Δ*kduD* mutant across multiple time points. The most severe defect, a reduction of 60.13%, was observed at 9 h. This phenotype was successfully complemented as biofilm formation was restored to near wild-type levels when the mutant was transformed with a plasmid carrying the *kduD* gene (pBAD24-*kduD*) (Figure 2I).

Further analysis using the Congo red-binding assay indicated that the deletion of *kduD* did not abolish the production of curli fimbriae and cellulose, which are key biofilm matrix components, and the colony morphology of the defined mutant was distinct from that of the initial transposon mutants (Figure 2J). Intriguingly, quantification of EPSs revealed a significant increase in EPS production in the *Ec*032Δ*kduD* mutant at equivalent bacterial densities (Figure 2K). These results demonstrated that *kduD* plays a critical role in mediating biofilm formation.

Collectively, these findings demonstrate that *kduD* is a key regulator of biofilm development in *Ec*032, likely through a mechanism involving the modulation of EPS production.

### 2.3. kduD Regulates Biofilm Formation by Modulating Flagellar Motility and EPS Production

**Figure 3:**
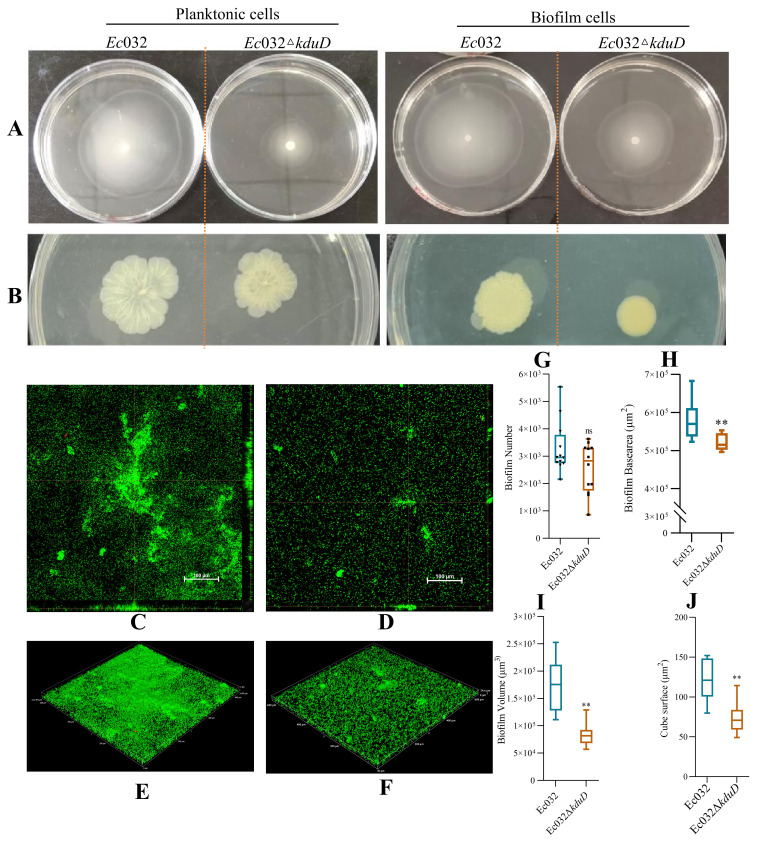
*kduD* regulates biofilm architecture by modulating flagellar motility and exopolysaccharide production. (**A**) Swimming zone diameter. (**B**) Twitching zone diameter. (**C**,**D**) Orthogonal views and three-dimensional representations of biofilms in the *Ec*032 group under 20× objective. (**E**,**F**) Orthogonal views and three-dimensional representations of biofilms in the *Ec*032Δ*kduD* group under 20× objective. (**G**) Biofilm cell number. (**H**) Biofilm base area. (**I**) Biofilm volume. (**J**) Surface area of biofilms. ** *p*  <  0.01.

Swimming and twitching motility assays were used to investigate the flagella and pili movement of the biofilm cells. The results showed that the swimming motility of the planktonic and biofilm cells of the *Ec*032Δ*kduD* strain decreased by 46.43% and 77.46% (Figure 3A), and the twitching motility decreased by 32.50% and 36.17%, respectively (Figure 3B). These results suggested that *kduD* mediates the formation of *E. coli* biofilms by regulating motility.

CLSM was utilized to observe the effect of *kduD* deletion on biofilm formation. As shown in Figure 2C–F, *Ec*032Δ*kduD* biofilms were significantly reduced compared to *Ec*032 biofilms. In addition, *Ec*032Δ*kduD* did not affect the cell number and base area (Figure 3G,H), but significantly reduced the volume and surface area of the biofilms (Figure 3I,J).

**Figure 4:**
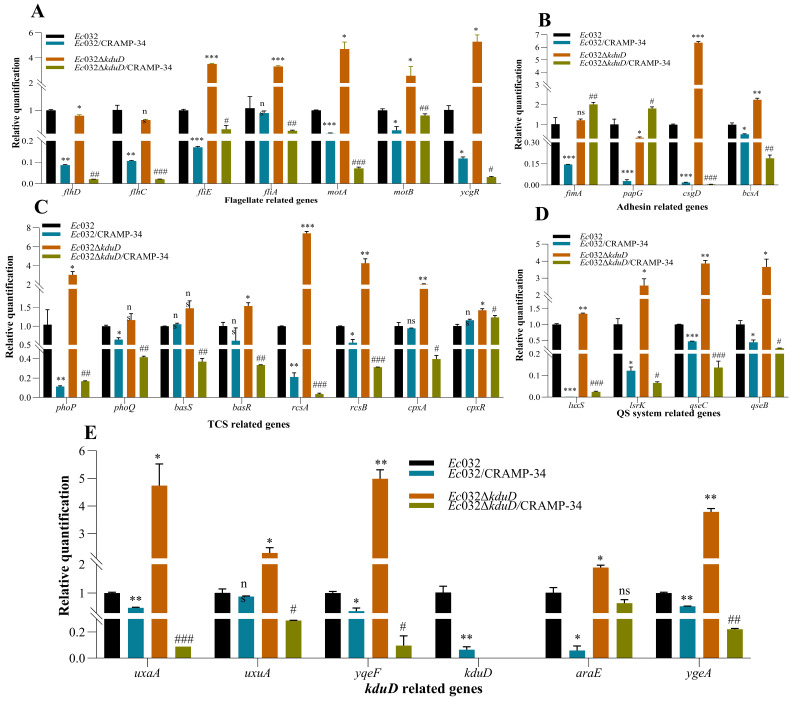
Relative expression of biofilm-associated genes. (**A**) mRNA levels of flagellate-related genes. (**B**) mRNA levels of adhesin-related genes. (**C**) mRNA levels of QS system-related genes. (**D**) mRNA levels of TCS-related genes. (**E**) mRNA levels of *kduD*-related genes. Data are expressed as the mean ± SD of three independent experiments. Note: * *p* < 0.05, ** *p* < 0.01, *** *p* < 0.001 compared to untreated *Ec*032 group; # *p* < 0.05, ## *p* < 0.01, ### *p* < 0.001 compared to untreated *Ec*032Δ*kduD* group.

We found that the mRNA expression levels of the biofilm-related genes in *Ec*032Δ*kduD* were upregulated compared to *Ec*032, as assessed by RT-qPCR. These biofilm-related genes included flagella-related genes (*fliE*, *fliA*, *motA*, *motB*, and *ycgR*) (Figure 4A), adhesion-related genes (*csgD* and *bcsA*) (Figure 4B), QS-related genes (*luxS*, *lsrK*, *qseC*, and *qseB*) (Figure 4C), TCS-related genes (*phoP*, *rcsA*, *rcsB*, and *cpxR*) (Figure 4C), and *kduD*-related genes (*uxaA*, *uxuA*, *yqeF*, *araE*, and *ygeA*) (Figure 4D). These results demonstrated that *kduD* plays a critical role in mediating biofilm formation.

Moreover, the mRNA expression levels of biofilm-related genes in CRAMP-34-treated *Ec*032 and *Ec*032Δ*kduD* were measured using RT-qPCR. The results showed that the mRNA levels of the flagella-related genes (*flhD*, *flhC*, *fliE*, *fliA*, *motA*, *motB*, and *ycgR*) (Figure 4A), adhesion-related genes (*csgD* and *bcsA*, but not *fimA* and *papG*) (Figure 4B), QS-related genes (*luxS*, *lsrK*, *qsEc*, and *qseB*) (Figure 4C), TCS-related genes (*phoP*, *phoQ*, *basR*, *rcsA* and *rcsB*, but not *basS* and *cpxR*) (Figure 4D), and *kduD*-related genes (*uxaA*, *uxuA*, *yqeF*, *kduD*, *araE*, and *ygeA*) (Figure 4E) were downregulated in *Ec*032 and *Ec*032Δ*kduD* after treatment with CRAMP-34. These results indicated that the scavenging effect of CRAMP-34 on mature biofilms of *E. coli* is related to EPSs and the *kduD* metabolic pathway.

**Figure 5:**
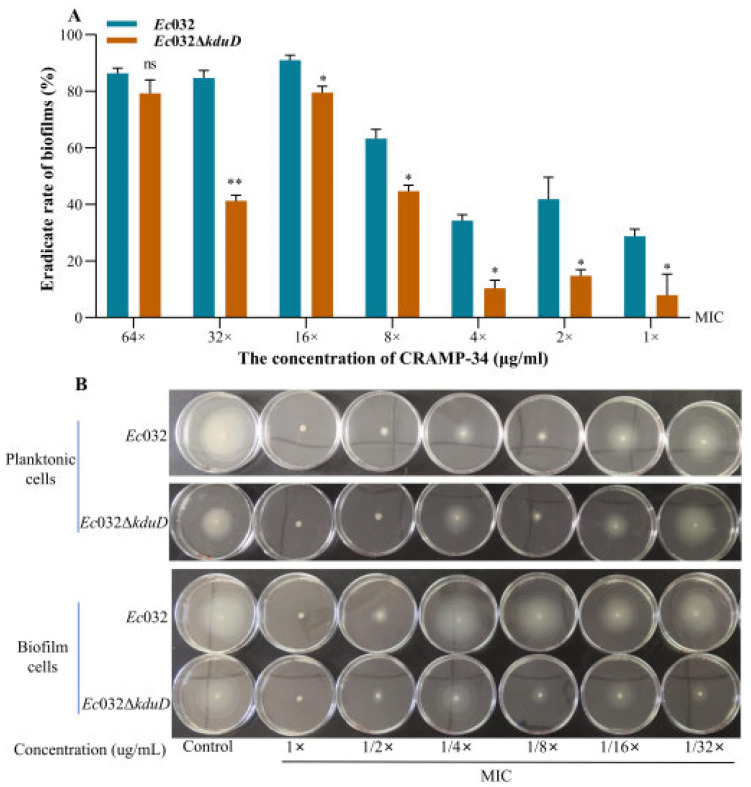
The biofilm inhibition rate of CRAMP-34 on 1-day-old preformed biofilms and the effect of CRAMP-34 on swimming motility. (**A**) The biofilm inhibition rate of CRAMP-34. (**B**) The swimming zone diameter (mm) after CRAMP-34 inhibition of planktonic and biofilm bacteria’s swimming motility. * *p*  <  0.05 and ** *p*  <  0.01.

The results of the crystal violet staining revealed that the biofilm inhibition rate of CRAMP-34 on *Ec*032Δ*kduD* was lower at all concentrations compared to *Ec*032 (Figure 5A). The effect of CRAMP-34 on the flagella motility of *Ec*032 and *Ec*032Δ*kduD* was observed by spotting planktonic and biofilms cells in liquid onto agar plates containing different concentrations of CRAMP-34. The results showed that CRAMP-34 inhibited the flagellar movement, which was dependent on the concentration of CRAMP-34 (Figure 5B).

## 3. Discussion

*E. coli* biofilms represent a serious public and animal health concern, yet the regulatory mechanisms governing their development remain incompletely elucidated. Our data demonstrate that the antimicrobial peptide CRAMP-34 effectively eliminates mature biofilms formed by pathogens such as *Pseudomonas aeruginosa*, *Acinetobacter baumannii*, and *E. coli*, while the present study elucidates the specific molecular mechanism of CRAMP-34 against a model *E. coli* biofilm; however, the full scope and clinical relevance of CRAMP-34 require further investigation in diverse infection models [15,18]. The observed requirement for relatively high concentrations (≥16× MIC) of CRAMP-34 to eradicate mature biofilms reflects both the intrinsic tolerance mechanisms of biofilms and the peptide’s unique mode of action. Unlike CIP, which primarily inhibits DNA replication, CRAMP-34 operates through membrane disruption combined with metabolic interference, specifically targeting the *kduD*-dependent network that is essential for biofilm maturation. This dual mechanism likely underlies its superior efficacy against established biofilms compared to conventional antibiotics. Although effective concentrations are higher under in vitro conditions, studies on CRAMP family peptides have demonstrated favorable safety profiles with low cytotoxicity and immunomodulatory potential at relevant doses, supporting their therapeutic feasibility [18]. Importantly, such concentration requirements can be achieved with localized sustained-release delivery systems (e.g., hydrogels and advanced dressings), which can maintain effective drug levels at infection sites while minimizing systemic exposure [19]. Structural advantages over human LL-37, including optimized hydrophobicity and helical stability, may enhance CRAMP-34′s penetration through biofilm matrices and interaction with bacterial membranes. In addition, CRAMP-34 significantly accelerated the healing of *Escherichia coli*-infected wounds in mice. This therapeutic effect can be primarily attributed to its direct antimicrobial activity, as evidenced by the marked reduction in bacterial load at the wound site. Given its potent anti-biofilm efficacy against mature biofilms in vitro, it is reasonable to infer that CRAMP-34 may also contribute to wound healing by disrupting biofilm structures in vivo, thereby enhancing bacterial clearance. The accelerated wound closure may partly result from the potential immunomodulatory functions of CRAMP-34 as a host defense peptide, which could indirectly promote healing by modulating local inflammatory responses or facilitating tissue repair. It should be noted, however, that the present study primarily focused on validating its antimicrobial and anti-biofilm effects. The specific mechanisms and relative contribution of its immunomodulatory role need to be further elucidated through follow-up experiments. Collectively, these properties position CRAMP-34 as a promising next-generation anti-biofilm agent, with its multi-mechanistic action, compatibility with targeted delivery approaches, and demonstrated efficacy supporting its potential for clinical translation to treat persistent biofilm-mediated infections.

To identify key the genetic determinants of biofilm formation and assess whether CRAMP-34 targets these pathways, we performed a transposon mutagenesis screen. All 25 isolated biofilm-deficient mutants mapped to *kduD*, showing that deletion of *kduD* severely compromised biofilm formation. As *E. coli* forms air–liquid interface biofilms—a process dependent on oxygen, flagellar motility, and cellulose—and given the established role of flagellar function in approximately half of *E. coli* biofilms mutants [20,21], we investigated the motility of *Ec*032Δ*kduD*. The mutant showed markedly impaired swimming motility despite the upregulation of flagellar assembly (*fliE* and *fliA*), motor genes (*motA* and *motB*), and the c-di-GMP-responsive brake protein gene *ycgR*. Since *YcgR* binds to c-di-GMP to mechanically inhibit flagellar rotation, its overexpression likely explains the motility defect [22]. Furthermore, the upregulation of the flagellar regulatory system *qseBC* suggests that *KduD* functions within or downstream of the *QseBC* circuit, potentially influencing motility via c-di-GMP and *YcgR* [23]. In summary, *KduD* emerged as a novel essential regulator of biofilm formation in *Ec*032, likely through the *QseBC* system and a c-di-GMP–*YcgR*-mediated braking mechanism. Its exact molecular role and therapeutic relevance as a target of CRAMP-34 warrant further study.

The established model of biofilm development proceeds through attachment, microcolony formation, and maturation into complex structures such as mushroom-like macrocolonies. We observed that the *Ec*032Δ*kduD* mutant retained the ability to adhere and form microcolonies but failed to develop architecturally mature biofilms. This defect occurred despite the upregulation of the key matrix genes *csgD* and *bcsA* and a measurable increase in EPSs under a standardized bacterial density [24,25]. Notably, while cellulose and curli fibers—encoded by *bcsA* and *csgD*, respectively—are essential for structural integrity, particularly under nutrient limitations; their overproduction can disrupt surface adhesion and aggregation [26,27]. In *E. coli* MG1655, overexpression of *csgD* was shown to inhibit biofilm formation on hydrophobic surfaces due to excessive cellulose production [28]. Similarly, our findings demonstrate that deletion of *kduD* leads to a significant increase in total EPS production and alters colony morphology, indicating its essential role in biofilm matrix regulation. However, we recognize that the proposed link between *KduD*-mediated metabolism and biofilm matrix organization remains largely inferential as the composition of EPSs was not directly characterized in this study. We explicitly state that while *kduD* clearly influences EPS accumulation, the specific metabolic pathways and compositional shifts underlying this phenotype warrant further investigation. Future studies including EPS profiling, metabolomic analysis, and high-resolution imaging of the biofilm matrix will be essential to elucidate the precise mechanism through which *kduD* coordinates EPS synthesis and biofilm architecture. In support of this, we observed concomitant upregulation of the RcsAB, a known repressor of *csgD*, likely reflecting a compensatory cellular response to restore regulatory balance [29,30]. Thus, *KduD* appears to act as a metabolic modulator whose activity fine-tunes matrix biosynthesis, ensuring the appropriate spatial and temporal deposition of the polysaccharide and protein components required for a stable biofilm architecture [31].

*KduD*, a 2-dehydro-3-deoxy-D-gluconate 5-dehydrogenase, participates in pentose and glucuronate metabolism [32]. Our analysis revealed that deletion of *kduD* triggered upregulation of the adjacent genes *ygeA* and *yqeF*, which are involved in glucose metabolism, indicating a compensatory metabolic adaptation. Given that *KduD* is involved in the metabolism of galacturonic and glucuronic acids [33]—key precursors for EPS biosynthesis—we hypothesize that it regulates biofilm architecture by controlling the supply of essential carbon substrates. This is supported by the observation that the *Ec*032Δ*kduD* mutant, similar to colanic acid (CA)-deficient strains, failed to form mature biofilms with the typical mushroom-like structure. CA, a branched EPS composed of glucose, galactose, and glucuronic acid, is critical for building the three-dimensional matrix of *E. coli* biofilms [34]. The inability to form structured biofilms may thus stem from a shortage of these vital EPS building blocks, impairing bacterial colonization and survival in the mammalian intestine. Furthermore, the upregulation of *ygeA*, which encodes an amino acid racemase that produces biofilm-dispersing D-amino acids, may represent a concurrent disassembly signal that further destabilizes the biofilm matrix [35,36]. In conclusion, we propose that *KduD* influences biofilm formation not through direct regulatory means, but by being a critical metabolic node that fuels the biosynthesis of structural EPSs, thereby linking central carbon metabolism to biofilm structural integrity.

Our data demonstrate that CRAMP-34 exerts potent anti-*Ec*032 biofilm activity by inhibiting flagellar motility and eradicating mature biofilms. The observation that *kduD* expression is drastically suppressed following CRAMP-34 treatment prompted us to investigate a potential functional link. The finding that CRAMP-34′s *Ec*032Δ*kduD* biofilm clearance efficacy was significantly attenuated, though not entirely abolished, provides direct genetic evidence that *KduD* is a critical target for its action. This partial persistence of activity suggests that CRAMP-34, like many effective antimicrobial peptides, employs a polypharmacological strategy, engaging multiple cellular targets to disrupt the complex biofilm network. The RT-qPCR analysis results supported this model by revealing a marked downregulation of the *kduD*-associated metabolic operon (which includes *uxaA* and *uxuA*) and the glucose transporter *araE* upon CRAMP-34 treatment. This indicates a targeted disruption of a specific metabolic pathway centered on *KduD*. Furthermore, the significant suppression of the key biofilm regulators *csgD* and *bcsA* aligns with the proposed role of *KduD* in maintaining EPS stability and suggests a cascade of transcriptional repression. The temporal disconnect between this rapid transcriptional reprogramming and the subsequent phenotypic collapse is a hallmark of targeted anti-virulence strategies, where disabling master regulators leads to a progressive dismantling of the biofilm structure.

## 4. Materials and Methods

### 4.1. Bacterial Strains, Plasmids, Primers, and Growth Conditions

*E. coli* (No. Ec032) was isolated from porcine sources and identified as a colistin-resistant strain carrying the mcr-1 gene of the plasmid IncX4 with a strong biofilm formation ability [1]. The bacteria were grown overnight at 37 °C in Brain Heart Infusion (BHI) broth (Haibo Co., Ltd., Qingdao, China), harvested by centrifugation at 6000 rpm for 10 min, and resuspended in BHI broth. The optical density of the bacterial suspension was measured at 600 nm (OD600 nm). This suspension was used in the subsequent experiments. The strains, plasmids, and primers are listed in Table 1, Table 2 and Table 3, respectively.

### 4.2. Determination of Minimum Inhibitory Concentration (MIC)

The MIC values of the antibiotics (ciprofloxacin, CIP; Yuanye Co., Ltd., Shanghai, China) and antibacterial peptides (AMPs; CRAMP-34 and LL-37; synthesized by ChinaPeptides Co., Ltd., Shanghai, China) were determined using the microbroth dilution technique described by the Clinical and Laboratory Standards Institute (CLSI).

### 4.3. Biofilm Formation and Anti-Biofilm Assays

For the microtiter plate test (in 96-well plates), mature biofilms were formed by adding 100 μL the test bacterial solution into each well of the 96-well plates and incubating them at 37 °C for 24 h. Then, the plates were washed with phosphate-buffered saline (PBS). Two-fold dilutions of CRAMP-34, LL-37, and CIP ranging from 64 to 0.5× MIC were prepared with sterile water. Next, each antimicrobial compound was added to the corresponding plate and incubated at 37 °C for 3 h. The appropriate concentration of CRAMP-34 was selected and tested in a 6-well plate (Corning^®^3516, Corning Incorporated, Corning, NY, USA) to confirm the results; PBS was used as a control. For selective growth of the bacteria, the following antibiotics and substances were added: 2 μg/mL colistin, 30 μg/mL apramycin, 100 μg/mL rifampin, 57 μg/mL diaminopimelic acid, 5% sucrose, and 2 mg/mL L-arabinose.

### 4.4. Skin Wound Infection Model Was Established

The animal experiments utilized male Balb/c mice (6–8 weeks old, weighing 20–23 g; Dashuo Co., Ltd., Chengdu, China). All housing, handling, and experimental procedures strictly followed the national standard GB/T 35823-2018; Laboratory Animal-Guidelines for Ethical Review of Animal Welfare (National Standards of the People’s Republic of China; Beijing, China, 2018). The protocol was approved by the Institutional Animal Care and Use Committee of Southwest University (Permit No.: LAC2025-1-0259). Initially, the mice were subjected to general anesthesia. Subsequently, a 1 cm diameter artificial wound was created on the dorsal surface of each mouse using surgical scissors. A volume of 200 µL of bacterial suspension (108 CFU/mL) was applied topically to the wound immediately post-injury and allowed to colonize for 24 h prior to the initiation of treatment. After successful establishment of the skin infection model, the mice were randomly assigned to two groups (n = 10 per group): the PBS treatment control group that was infected with the Ec032 strain and the CRAMP-34 treatment group that was infected with the Ec032 strain. The treatment was administered as a single topical application on day 1 post-infection. A dose of 50 µg/wound of CRAMP-34 (dissolved in PBS) or an equal volume of PBS was applied directly to the wound surface. No further treatment was administered on the subsequent days. The total observation period was 7 days post-infection. Throughout the treatment period, wound changes were documented daily via photography, and samples were collected for bacterial counting.

### 4.5. Crystal Violet Staining and Colony Count Assay

The biofilm formation assay was performed using crystal violet, and the viable bacteria were counted on trypticase soy agar plates. Briefly, the supernatant was discarded, and the cells were washed twice with sterile PBS, fixed in methanol for 10 min, and stained with crystal violet for 20 min. After washing, acetic acid was used to dissolve the bound crystal violet, and the absorbance was measured at OD600 nm. To count the number of bacterial biofilm cells, 100 µL of Triton-100X was added to disrupt the biofilms, and the bacteria were spread on TSA plates at a 10-fold dilution at 37 °C for 12 h.

### 4.6. Swimming Motility Assay

LB medium plates with 0.25% (wt/vol) agar were used for the swimming motility assay. One-microliter aliquots of mid-log-phase bacteria in BHI broth were spotted onto a control agar plate and an agar plate containing decreasing concentrations of CRAMP-34. The diameters of the swimming zones were measured after incubation at 37 °C for 15 h.

### 4.7. Confocal Laser Scanning Microscopy (CLSM)

The morphological features of biofilms were observed using CLSM as described previously with some modifications [1]. In this experiment, 500 µL of the test bacterial solution (OD600 nm = 0.1) was added to an 8-well chambered coverglass (Lab-Tek II, Rochester, NY, USA), and the medium was replaced every 24 h. After incubation at 37 °C for 2 days, the biofilms were treated with CRAMP-34 (125 μg/mL) at 37 °C for 3 h, washed with 0.9% (wt/vol) NaCl, and stained at room temperature for 20 min in the dark using a Filmtracer™ LIVE/DEAD™ Biofilm Viability Kit. After rinsing with sterile water, the biofilm samples were imaged with a point-scanning confocal microscope (Eclipse Ti2; Nikon, Tokyo, Japan). The signals were recorded using the green (SYTO9, excitation wavelength of 488 nm) and red (PI, excitation wavelength of 561 nm) channels. The field of view under the ×20 objective was randomly selected, and a three-dimensional image was constructed by stacking multiple images with different Z values (z-stack). The images were acquired using NIS Viewer v5.21.00 software. Four representative images were selected from each biofilm, and each experiment was repeated at least three times. The biofilm-related test parameters, such as the cell number, volume, base area, and fluorescence intensity, were analyzed using BiofilmQ software version 2.1.0 (The authors, Vienna, Austria).

### 4.8. Screening of Gene Mutants with Decreased Biofilm Formation

For the primary screening of the transposon library, the transconjugants were cultured to the mid-log phase, and then 5 μL was added to Corning 3595 microtiter plates containing 100 μL of BHI broth and incubated at 37 °C for 24 h. The mature biofilms were assessed by crystal violet staining. Isolates exhibiting reduced biofilm accumulation (<75% of the wild-type level) in the primary screen were retested individually, as described previously [1]. For all mutants with a confirmed defect in biofilm accumulation, the growth kinetics were evaluated based on the changes in the bacteria in BHI broth over time at OD600 nm. The formation of biofilms is generally divided into four grades based on the average OD value of the negative control (ODc): OD ≤ ODc is non-adherent, ODc < OD ≤ 2× ODc is weakly adherent, 2× ODc < OD ≤ 4× ODc is moderately adherent, and 4× ODc < OD is strongly adherent.

### 4.9. Generation of a Ec032 Mutagenesis Library

*E. coli* WM3064 (pCure-oriT-GFP-MCR) and *Ec*032 were used as the donor (carrying Mariner transposons with a GFP gene) and the recipient, respectively. After the donor and recipient bacteria were cultured to the logarithmic growth stage, they were conjugated at 37 °C at a ratio of 1:1 for 8–12 h. The transconjugants with GFP were selected on LB agar containing 30 μg/mL apramycin and induced in sugar-free LB broth (Yuanye Co., Ltd., Shanghai, China) with 2 mg/mL L-arabinose (Shenggong Co., Ltd., Shanghai, China) for 6 h to obtain a plasmid-free *Ec*032 strain containing the pCure-oriT-GFP-MCR plasmid. These strains were cultured on LB agar plates containing 5% sucrose (Maclin Co., Ltd., Shanghai, China) at 37 °C for 24 h to obtain a plasmid-free *Ec*032 strain (*Ec*032Δ). *E. coli* WM3064 (pCat-arr) (donor carrying Mariner transposons) and *Ec*032Δ (recipient) were conjugated at 37 °C at a ratio of 1:1 for 8–12h. The transconjugants were obtained in a total volume of 1 mL. The transconjugants were selected on LB agar containing 100 μg/mL rifampin to positively select for arr transconjugants. A 100 μL aliquot was diluted to a suitable gradient and used to coat a plate; the number of mutants was calculated and a transposon mutation library was obtained.

### 4.10. Identification of Transposon Insertion Sites

ERIC-PCR was used to detect any contamination of the screened transposon mutants with decreased biofilm formation. The genomic DNA of Ec032 and the transposon insertion mutants with reduced biofilm formation was extracted using the TaKaRa MiniBEST Bacteria Genomic DNA Extraction Kit Ver. 3.0 (TaKaRa Co., Ltd., Beijing, China) and subjected to whole-genome sequencing at Novogene Co., Ltd., Beijing China. The insertion site of the Mariner transposon was identified using SnapGene software and verified using PCR validation with the primers Ec032-F-XJ and Ec032-R-XJ. The sequence was compared to the NCBI database to identify the gene name and base sequence for the subsequent gene editing.

### 4.11. kduD Mutant Construction Using CRISPR/Cas9 System

Plasmids and genomic DNA were extracted using the TaKaRa MiniBEST Bacteria Genomic DNA Extraction Kit Ver. 3.0 (Takara Biomedical Technology (Beijing) Co., Ltd., Beijing, China). In this study, we used two plasmid systems (which express Cas9 and the sgRNA in pCasKp-OriT and pSGKp, respectively) for genome editing using the CRISPR-Cas9 system in *E. coli* DH5α. First, pSGKp-*Ec*032-*kduD*, which encodes the sgRNA targeting the *kduD* gene (target sequence selected by SnapGene software), was constructed. Briefly, the guide RNA target sequence in pSGKp was mutagenized using PCR to target *kduD*. Primers *kduD*-F1, *kduD*-R1, *kduD*-F2, and *kduD*-R2 were used to amplify left homologous arm H1 and right homologous arm H2. The pSGKp-*Ec*032-*kduD* plasmid was cut into linear DNA using BamHI and XbaI enzymes. The left and right homologous arms H1 and H2, and linear pSGKp-*Ec*032-*kduD* plasmids were connected using the NEBuilder HiFi DNA Assembly Master Mix (NEB Biological Technology (Beijing) Co., Ltd., Beijing, China) and then transformed into *E. coli* DH5α to obtain pSGKp-*Ec*032-*kduD*-500 plasmids. We performed cloning for a pSGKp-*Ec*032-*kduD*-500 plasmid harboring a spacer (for guiding Cas9 cleavage of the wild-type *Ec*032) in *E. coli* DH5α, which was selected on LB plates with 100 μg/mL rifampin (Shanghai Maclin Biochemical Technology Co., Ltd., Shanghai, China). The transformants were confirmed by sequencing.

*E. coli* WM3064 (pCasKp-OriT) and *Ec*032 (pSGKp-*Ec*032-*kduD*-500) were used as the donor and recipient, respectively. After conjugation, the plasmid pCasKp-OriT from *E. coli* WM3064 was introduced into *Ec*032. The transconjugants were selected on LB agar containing 30 μg/mL apramycin (Shanghai Maclin Biochemical Technology Co., Ltd., Shanghai, China) at 30 °C for 8 h–12 h. Then the transconjugants were confirmed by colony PCR and DNA sequencing. The gene deletion strains were plated onto 5% sucrose LB agar plates and cultured at 37 °C to eliminate plasmids pSGKp-*Ec*032-*kduD*-500 and pCasKp-OriT.

### 4.12. Obtaining Ec032ΔkduD Plasmid Deletion Strains

The construction of *kduD* complementation strains followed the design for plasmid pBAD24. The *kduD* gene fragment, amplified from the genomic DNA of *Ec*032, was inserted into the multiple cloning site (MCS) of plasmid pBAD24 using the ClonExpress^®^ Ultra One Step Cloning Kit (Vazyme Biotech Co., LTD, China). Then, the recombinant product pBAD24-*kduD* was transformed into *E. coli* DH5α chemically competent cells and inoculated on transformed LB plates supplemented with 100 μg/mL ampicillin. Positive clones were identified through colony PCR and sequencing. Finally, the recombinant plasmid was transferred into a *kduD* mutant strain via electroporation. The constructed complementation strain, named *Ec*032-Δ*kduD*/p*kduD*, was induced with arabinose during culturing and was used to ascertain the target of CRAMP-34 in *Ec*032.

### 4.13. Growth Curves

The bacteria were cultured in BHI broth to the mid-log phase and diluted to equal optical densities (OD600 nm = 1). A 200 µL volume of these cultures was added to 96-well microtiter plates (Corning^®^3599; Corning Inc., Corning, NY, USA) and incubated at 37 °C. The growth of these cultures was monitored by determining the absorbance at 600 nm every 2 h for 24 h. The OD600 nm was measured using an Infinite^®^ M Plex microplate reader (Tecan, Switzerland) after shaking. Three independent experiments were performed.

### 4.14. Congo Red-Binding Assay and EPS Assay

One microliter of the test bacterial suspensions of *Ec*032 and *Ec*032Δ*kduD* was spotted onto 40 μg/mL Congo red salt-free LB agar plates, which were incubated at 37 °C for 2–4 days. Subsequently, their colony morphologies were compared.

EPSs were determined using the phenol–sulfuric acid method. The working bacterial suspension was inoculated into 6-well plates and cultivated at 37 °C for 24 h to form biofilms. Then, the planktonic bacteria were discarded, the wells were rinsed with PBS, normal saline was added to scrape up the biofilms, which were homogenized by repeated and even blowing. The resulting suspension was clarified by centrifugation at 4000 rpm at 4 °C for 20 min. The supernatant was filtered through a 0.22 μm filter membrane; 100 μL was added into a new centrifuge tube and mixed with 200 μL of concentrated sulfuric acid and then 25 μL of a 6% phenol solution in a water bath at 90 °C for 5 min. After cooling to room temperature, 200 μL was dispensed into a 96-well plate to measure the absorbance at the wavelength of 490 nm. Each experiment was set up with three parallel groups and was repeated three times.

### 4.15. Real-Time Fluorescence Quantitative PCR (RT-qPCR)

The CRAMP-34 treatment of the biofilms was carried out as described above. Total RNA was extracted using the Total RNA Isolation Reagent (Biosharp Co., Ltd., Beijing, China) according to manufacturer’s instructions. RNA was reverse transcribed using the PrimeScript™ RT reagent Kit (Takara Bio Inc., Shiga, Japan)according to the manufacturer’s instructions. Primers and probes were designed using Primer 5.0 and synthesized by Tsingke Biotechnology (Beijing, China). RT-qPCR was conducted on a CFX Connect qPCR instrument. The reaction conditions were as follows: 95 °C for 30 s and 40 cycles of 95 °C for 5 s and 60 °C for 30 s. The relative expression levels of each target gene were computed using the 2−ΔΔCt method.

### 4.16. Statistical Analysis

All experiments were conducted in triplicate, and the data represent a minimum of three biological replicates. The statistical analyses were carried out using Microsoft Excel and GraphPad Prism 8.0 software. An unpaired *t*-test (two-tailed) was used to calculate the statistical significance. Significant differences are indicated as * *p*  <  0.05, ** *p*  <  0.01, and *** *p*  <  0.001.

## 5. Conclusions

This study demonstrated that CRAMP-34 can effectively eradicate mature *E. coli* biofilms through mechanisms involving suppression of flagellar motility and disruption of biofilm structural integrity. We further identified *kduD* as a previously unrecognized key regulatory gene controlling bacterial motility and biofilm development. While the experiments primarily focused on 1-day-old biofilms—a standard model for assessing early biofilm susceptibility—the persistent role of *kduD*-dependent metabolic regulation throughout biofilm maturation suggests broader relevance to more established biofilms. Collectively, these findings not only highlight the therapeutic potential of CRAMP-34 as a dual-function antimicrobial and anti-biofilm agent, but also establish *kduD* as a central metabolic regulator in biofilm formation, offering a promising new target for innovative anti-biofilm strategies.

## Figures and Tables

**Table 1:** Bacterial strains and plasmids used in this study.

Strain/Plasmid	Description
*Ec*032	Clinical isolate of IncX4 plasmid strain carrying *mcr*-1, Colr
*Ec*032Δ	Plasmid was cured
*Ec*032Δ*kduD*	*kduD* gene deleted
*E. coli* DH5α	Cloning vectors
*E. coli* WM3064	A diaminopimelic acid (DAP) auxotroph strain
pCure-oriT-GFP-MCR	Aprr, oriT+, GFP+, sacB+
pCat-arr	Mariner transposon, Rifr, oriT+
pCasKp-OriT	Aprr, oriT+, bacterial expression of Cas9 nuclease and λ-Red recombination system with temperature-sensitive replication
pSGKp-arr2	Rifr, sacB+, sgRNA expression plasmid for targeting a specific sequence
pSGKp-*Ec*032-*kduD*	pSGKp derivative with the spacer of the *kduD* gene
pSGKp-*Ec*032-*kduD*-500	pSGKp derivative with the repair arms of the *kduD* gene

Note: Aprr, Apramycin-resistant; Rifr, Rifampicin-resistant; Colr, Colistin-resistant; oriT+, with conjugation transfer initiation site; sacB+, with the suicide gene sacB; GFP+, with GFP gene.

**Table 2:** Primers used in this study.

Primer	Sequence (5′-3′)
*Eric*-F	ATGTAAGCTCCTGGGGATTCAC
*Eric*-R	AAGTAAGTGACTGGGGTGAGCG
*Ec*032-F-XJ	GTCGAGAATTTCCGCGCTAC
*Ec*032-R-XJ	GTATTGATACCGGCACTCCG
infu-*kduD*-N20F	CACATAATCTGAAGCGCTGGGTTTTAGAGCTAGAAATAGCAAGTTAAAATAAGGC
infu-*kduD*-N20R	CCAGCGCTTCAGATTATGTGACTAGTATTATACCTAGGACTGAGCTAGC
*kduD*-N20-F	CACATAATCTGAAGCGCTGG
*kduD*-F1	TCGAATTCCTGCAGCCCGGGGGATCCTATTTACGCCCCAGGCGGAA
*kduD*-R1	CCGCATGGCAGGGTTTTTTA
*kduD*-F2	TAAAAAACCCTGCCATGCGGTATGCCCACAACTAGCGCAA
*kduD*-R2	TCCACCGCGGTGGCGGCCGCTCTAGATTACTGTCGATGGCCAATGC
*kduD*-F	TGCCGAGTGTGACCATCAAC
*kduD*-R	CGGAGGTTGATGTGGACGTA

**Table 3:** Primers for RT-qPCR.

Gene	Forward Primer Sequence	Reverse Primer Sequence
*gapA*	GTTGTCGCTGAAGCAACTGG	CGATGTCCTGGCCAGCATAT
*flhC*	ATGCTGCCATTCTCAACCGA	GCTTGTGGGCACTGTTCAAG
*fliE*	GACCATTAGTTTTGCCGGGC	ACGCACCTGAATCCCCATTT
*fliA*	GAACGCTATGACGCCCTACA	TCCAGTTGCCCTATTGCCTG
*motA*	CGCCGAAACCAGCAAAATGA	TCCTCGGTTGTCGTCTGTTG
*motB*	TGACTGCGATGATGGCCTTT	CCCCTGGCTTTGGGTGTAAT
*ycgR*	GGGGCAATGGGGTGTTTTTC	CTTTGTCCGCTTTTTCCCGG
*fimA*	TTGTTCTGTCGGCTCTGTCC	ACTGGTTGCTCCTTCCTGTG
*papG*	TTCGCATCGTGAAACAGCAC	TACGTTTCGCTTCCATGGCT
*csgD*	GATTACCCGTACCGCGACAT	GCGTAATCAGGTAGCTGGCA
*bcsA*	AACGAAGGCACGCTGTTCTA	GAGGTATAGCCACGACGGTG
*luxS*	TTGGTACGCCAGATGAGCAG	ACGTCACGTTCCAGAATGCT
*lsrK*	CAGATTACTTTGGCTGGCGC	CGTAGGCCAGCCATATCCAG
*qseC*	CGTGACCCTGACTCGGAAAA	TTCGGTTTGCACTTTCAGCG
*qseB*	ATTGGCGACGGCATCAAAAC	CACGCTGACCTTTTTCTCGC
*phoP*	GCGCGTACTGGTTGTTGAAG	CTGGCAATCCGAGATCGACA
*basS*	CCTGCTGCGGATGTTATTGC	ACGCTTTACTCAACTCCCCG
*basR*	GTACTGATCCTCACCGCTCG	GACCCATGTTCAGCGTCAGA
*rcsA*	ATTGAGCCGAACCGAATCGA	GTCAGTCGGACGACATGGTA
*rcsB*	ATCAGTGCTGGTGGTTACGG	TCAGCAGGGCGATATCGTTC
*cpxA*	CGCAGGTGCCAGTTTTAACC	CAGTTCCTTGCTTTCACCGC
*cpxR*	TTAGTGCTGAATCCAGGCCG	CGTTTGCCCAACACTTCCTG
*kduD*	AACCATCGAGCAGGTCACAG	TCGCTGAACTCGAGAGCATC
*uxuA*	ACCAGATCGAATTCGCTGCA	CCCGGAAGACCAGCAATGAT
*uxaA*	GTGATTGGTCTGGGCTGTGA	CTGGCTCGCGTTTATCGTTG
*araE*	TTACCTGTTCGACCACCACG	GGTTTCCGGAATGAGCCAGA
*ygeA*	ACGATGCATAAAGTGGCGGA	AAAATTGTTCCGTCAGCCGC
*yqeF*	TTGCCAGCGTTGGTGTAGAT	ATTGACATTGACCCGACGCT

## Data Availability

The original contributions presented in the study are included in the article; further inquiries can be directed to the corresponding author.

## Abbreviations

The following abbreviations are used in this manuscript:

*E. coli*	*Escherichia coli*
CRAMP-34	cathelicidin-related antimicrobial peptide
EPS	exopolysaccharides
AMP	antibacterial peptide
PCR	polymerase chain reaction
QS	quorum sensing
TCS	two-component system
c-di-GMP	cyclic diguanosine monophosphate
*KduD*	2-dehydro-3-deoxy-D-gluconate 5-dihydrogenase
CIP	ciprofloxacin
MIC	minimum inhibitory concentration
CLSM	Confocal Laser Scanning Microscopy
RT-qPCR	real-time fluorescent quantitative PCR
